# Acute treatment of elderly patients with acetabular fractures by open reduction, internal fixation, and total hip arthroplasty: a 1–10-year follow-up of 48 patients

**DOI:** 10.2340/17453674.2024.42113

**Published:** 2024-11-20

**Authors:** Ragnhild Loven KIRKEBOE, Jan Erik MADSEN, Lars NORDSLETTEN, John CLARKE-JENSSEN

**Affiliations:** 1 Division of Orthopedic Surgery, Oslo University Hospital, Oslo; 2 Institute of Clinical Medicine, University of Oslo, Oslo, Norway

## Abstract

**Background and purpose:**

Acetabular fractures in osteoporotic bone are associated with substantial joint impaction and comminution, previously shown to be prognostic for a poor result. A combined procedure of open reduction, internal fixation (ORIF), and total hip arthroplasty (THA) can be a good option, allowing for immediate weightbearing as tolerated. We report short- to medium-term outcome and complications of the results of patients treated with this combined procedure.

**Methods:**

48 cases treated with ORIF and acute THA from 2000 to 2019 were identified from our local pelvic fracture registry, from which follow-up data was extracted. Descriptive statistics were used and Kaplan–Meier survival curves were calculated. Primary outcome was HHS at 1 year. Secondary outcomes were implant survival, complications requiring surgery, and mortality at 3 months.

**Results:**

There were 37 men and 11 women treated in the study period. Mean age was 68 (37–87) years. 6 patients died within 3 months of surgery, leaving 42 cases available for follow-up. Mean follow-up (FU) was 2.8 (1–16) years. The most common mechanism of injury was fall from standing height (n = 36). Medical comorbidities were common. Mean Harris Hip Score (HHS) was 83 (51–100) at 1-year follow-up. There were 7 reoperations: 6 for postoperative infection and 1 closed reduction of implant dislocation. 38 had their implant intact at latest FU. At the latest FU, 28 patients were ambulatory without a walking aid.

**Conclusion:**

Our results indicate that ORIF and acute THA can be performed with good functional results in patients with unreconstructable displaced acetabular fractures, but with a significant risk of infection and revision.

The incidence of acetabular fractures in the elderly is increasing [1,2]. These acetabular fractures are often associated with severe joint impaction and comminution, factors shown to prognosticate poor results [3-5]. Anatomic reduction and stable fixation are essential for a favorable postoperative outcome [4]. This has proven challenging in osteoporotic bone [6]. Age has been cited as an important risk factor for conversion to THA after open reduction and internal fixation (ORIF) for displaced acetabular fractures [4,7]. The rate of conversion to THA after surgical treatment of an acetabular fracture is reported to be as high as 30%, with most cases converted within 2 years [3,8]. Previous studies have also included younger patients. It is therefore likely that a larger number of geriatric fractures treated by ORIF may develop an early need for THA, compared with younger patients. To reduce the complications of prolonged bedrest, elderly individuals are likely to benefit from a surgical solution allowing immediate full weightbearing. Thus, a joint-preserving procedure may not be the optimal choice of treatment for these patients.

A procedure combining ORIF and acute total hip arthroplasty (THA) has been described as a concept of an “A-frame,” stabilizing both columns and the acetabular shell acting as a horizontal column, which allows immediate weightbearing as tolerated [9]. Better clinical outcomes and fewer revisions with acute THA compared with delayed THA are also reported [10,11]. However, a significant risk of severe complications such as infection and dislocations indicates that patient selection is crucial to obtain good results [12,13].

In our institution, a combined hip procedure utilizing standard arthroplasty components, as reported by Rickman et al., has been used for unreconstructable acetabular fractures, characterized by severe joint comminution or impaction, and/or significant femoral head injury, femoral neck fracture, or severe osteoporosis [9]. We report on the short- to medium-term outcome and complications of this procedure and attempt to identify factors relevant for patient selection.

## Methods

Using our local pelvic fracture registry, patients treated with ORIF and acute THA were identified.

The main indications for the combined hip procedure were severe comminution, large dome impactions, femoral head injury and poor bone stock, defined as osteoporosis, previous fractures or radiographic features such as thinning of cortical bone, all factors indicating an unreconstructable hip joint (see Figure 2). In 2 cases, a simultaneous fracture of the proximal femur or femoral head provided the indication for the combined hip procedure.

An experienced pelvic trauma surgeon and a revision arthroplasty surgeon planned for the combined procedure. The surgical exposures were dictated by the fracture patterns. For fractures involving the anterior column and/or the quadrilateral plate, an anterior intrapelvic approach (AIP) was used and the fracture stabilized with plate(s), since 2016 with a QLS suprapectineal plate (Stryker GmbH, Selzach, Switzerland). For fractures through the posterior column and/or wall, the Kocher–Langenbeck (KL) approach and reconstruction plates were used. When a stable construct of both columns and walls had been achieved, the THA was implanted through a KL approach. Standard arthroplasty implants, used for primary and revision surgery in our department at the time, were used. In 1 case, a trabecular metal augment was used due to bone loss in the posterior wall. No other augments or cage constructs were used. Immediate weightbearing as tolerated was permitted.

The preoperative physiological status of each patient was assessed by an anesthesiologist using the ASA physical status classification system [14].

Fractures were classified using the Letournel classification of acetabular fractures [15].

The study is reported according to STROBE guidelines.

### Outcome

Follow-up data was prospectively collected in our pelvic fracture registry, a local registry in which all surgically treated pelvic and acetabular fractures are included by the pelvic surgeons. The standard protocol for follow-up in our registry was 3 months, 1, 2, 5, 10, 15, and 20 years. The baseline set of data included mechanisms of injury, fracture classification, type of surgery, and surgical exposures. At follow-up, complications were registered, clinical and radiographic assessments were performed, including pelvic radiographs, Harris Hip Score, WOMAC, and Merle D’Aubigne and Postel scores.

The primary outcome measure was HHS at 1-year follow-up. Secondary outcomes were implant survival, complications requiring surgical intervention and mortality at 3 months. We also aimed to identify possible risk factors for complications, such as medical comorbidities, fracture type, and duration of surgery by searching the electronic patient records.

Based on the findings in the current and previous studies, our institution follows a treatment algorithm for geriatric acetabular fractures (see Figure 4). Minimally displaced fractures are mainly treated nonoperatively with good results [16]. Displaced fractures without impaction or comminution can be treated with conventional ORIF.

### Statistics

Statistical analyses were performed using Stata SE (Stata Statistical Software, Release 16, 2019; StataCorp LLC, College Station, TX, USA). Descriptive statistics were used for the presentation of cases and outcome. Kaplan–Meier survival curves were calculated to show cumulative implant survival, with revision for any cause as endpoint. Due to loss to follow-up or deaths, the number left at risk decreased after 2 years. Statistical significance was set at P < 0.05.

### Ethics, registration, data sharing, funding, use of AI, and disclosures

The study was approved by the institutional ethical review board (18/23992). The data was collected from our local pelvic fracture registry, and approved by the ethical review board for storing of data and for research purposes. We received no funding for this study. No artificial intelligence (AI) was used in analyzing data or preparing the manuscript. We report no conflict of interests. Complete disclosure of interest forms according to ICMJE are available on the article page, doi: 10.2340/17453674.2024.42113

## Results

**Figure 1:**
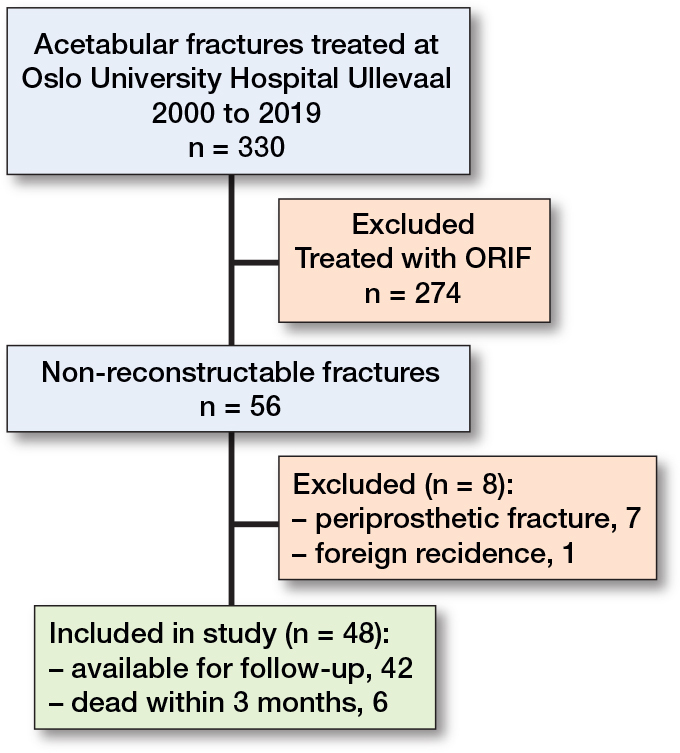
Flowchart of patients in the study.

From 2000–2019, 330 cases were treated for acetabular fracture. 274 were treated with ORIF only and were thus excluded; 56 fractures were deemed unreconstructable and considered for inclusion. 7 were excluded due to periprosthetic fracture and 1 was a tourist with foreign residence. 48 cases were therefore included in the study (Figure 1).

**Figure 2:**
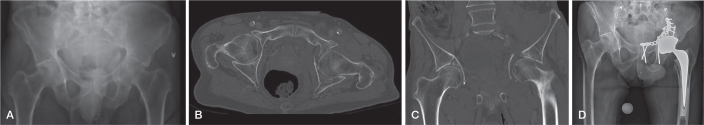
Preoperative radiography (A) of a typical fracture in a 75-year-old male with known hypertension and who was a smoker. He fell from standing height on an icy sidewalk. The fracture is a low anterior column fracture with medialization of the femoral head, and grossly displaced quadrilateral wall and joint. Axial (B) and coronal (C) CT scans confirm the findings. Postoperatively (D) the anterior column is reduced and stabilized with a suprapectineal plate through an anterior intrapelvic approach, the fracture repositioned, and the THA implanted through a posterior approach.

**Figure 3:**
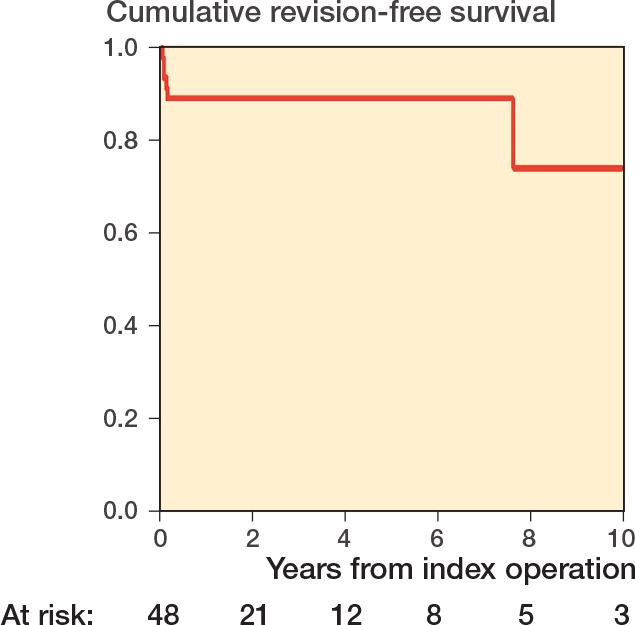
Cumulative survival of the THA over time in years, with revision of any cause defined as failure. The number of cases left at risk at each time interval is available at the bottom of the figure. The number at risk decreased at 2 years due to loss to follow-up and deaths after 1 year.

**Figure 4:**
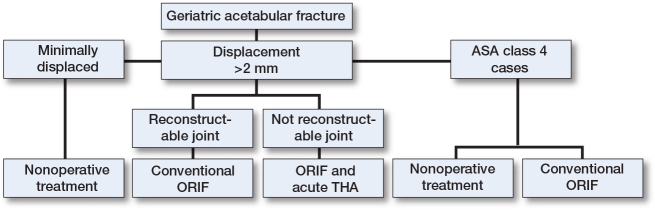
Proposed treatment algorithm for geriatric acetabular fractures for patients medically fit for surgery. More than 2 mm step in the joint is regarded as significant dislocation and indication for operative treatment. For patients deemed unfit for surgery or ASA class 4 patients, nonoperative treatment should be chosen, or, in select cases, conventional ORIF.

6 cases died within 3 months after surgery, leaving 42 cases available for follow-up. Complete clinical outcome data at 1 year was available for 36 cases. As verified by the ASA classification, all cases had at least 1 medical comorbidity, most commonly cardiovascular disease (n = 21) (Table 1). Mean follow-up was 2.8 (range 1–16) years. There were 37 men and 11 women with a mean age of 68 (range 37–87) years; the youngest patient in this series was biologically elderly with multiple medical comorbidities including osteoporosis. 6 patients were younger than 60 years, 3 of whom were polytrauma patients.

**Table 1:** Demographic details of the patients treated with ORIF and acute THA

Male/female	37/11
Mean age (range)	68 (37–87)
ASA classification	
2	13
3	15
4	6
Mechanism of injury	
Fall from standing	32
High-energy ^a^	13
Other ^b^	3
Polytrauma ^c^	5

a Fall from > 2 m, motor vehicle accidents, crush injuries, and motorcycle accidents.

b Bicycle or skiing injuries.

c Several concomitant fractures and/or abdominal or thoracic injury.

The most frequent mechanism of injury was a fall from standing height. 5 cases were polytrauma cases, all of whom had injuries to other organ systems and 4 had at least 1 additional extremity injury. 1 of the polytrauma cases had bilateral acetabular fractures. 3 of the polytrauma cases also sustained nerve injuries that were preoperatively recognized: 1 sciatic nerve injury due to a posterior fracture dislocation of the hip and 2 peroneal nerve injuries due to knee dislocation or tibia fracture.

The most common fractures were anterior column and anterior column posterior hemitransverse fractures (Table 2). There were 34 cases with significant dome impaction (Figure 2).

**Table 2:** Fracture classification according to the Letournel classification of acetabular fractures of the 48 acetabular fractures treated with the combined procedure

Type	n
Anterior wall	4
Anterior column	13
Posterior wall	7
Transverse	1
Transverse posterior wall	6
Anterior column posterior hemi transverse	13
Associated both columns	4

Mean duration of surgery was 220 minutes (range 117–330) for 12 cases treated with 1 surgical exposure, and 252 minutes (range 152–369) for 23 cases treated with 2 surgical exposures. For the remaining 13 cases, the duration of surgery could not be retrieved from the patients’ records. In cases with 2 approaches, the repositioning time was included in the total operating time. There was a reduction in duration of surgery during the study period; however, this was not statistically significant.

Several different hip implants were used, the most common being the Trabecular Metal (Zimmer Biomet) cup and a Corail (DePuy Synthes) stem (Table 3). The variety of implants used was due to changing practices in our department. All implants were routinely used for primary and revision arthroplasty during the study period.

**Table 3:** Implants used in the study

Type	n
Acetabular cup	
Trabecular Metal	30
Charnley Ogee ^a^	9
Avantage ^a^	3
Marathon ^a^	4
Exeter X3 ^a^	1
Femoral stem	
Charnley ^a^	13
Exeter ^a^	12
CPT ^a^	7
Corail	15

a Cemented.

The data was missing on 1 case that died before postoperative imaging was performed, and the implant type could therefore not be determined.

### Clinical outcome

Complete data for HHS was available for 36 cases at 1 year. Mean HHS at 1 year postoperatively was 83 (range 51–100) (Table 4). Of the cases with missing data, 1 was dead 18 months after surgery. The other 5 patients were lost to follow-up and did not respond when contacted. 4 patients reported a poor outcome with HHS <70, 1 was previously treated for a proximal femoral fracture, 1 suffered a nerve injury at the time of the injury, and 1 had several concomitant fractures after polytrauma.

**Table 4:** Clinical outcome at 1 year: Harris Hip score was available for 36 cases, the remaining 6 were contacted but did not respond

Factor	n
Mobilization	
Ambulatory without aid	19
1 or 2 crutches ^a^	7
Walker	1
Wheelchair/not ambulatory	4
Harris hip score (n = 36)	
Excellent (91–100)	17
Good (81–90)	8
Fair (71–80)	7
Poor (> 70)	4

a 1 case reported use of crutches prior to the injury due to unrelated causes and reported return to pre-injury function at the latest follow-up.

3 cases with a poor result suffered postoperative infection. 3 of the cases with a poor HHS sustained nerve injuries at the time of injury. Other cases with poor results had preexisting conditions such as ipsilateral arthritis in the knee or ankle, or neurological comorbidities, that affected gait and pain level.

19 patients were walking unaided and 7 were using crutches. 3 were dependent on a wheelchair; all 3 had been treated for postoperative infection. At the latest follow-up, 38 cases had the index THA intact.

### Complications

8 of the original 48 patients suffered 1 or more postoperative complications requiring surgical intervention (Table 5).There were 6 deep infections requiring at least 1 surgical debridement. 2 of the cases treated for postoperative infection died within 3 months of the index surgery. In 3 cases, the THA had to be removed to eradicate the infection; 2 of these patients also had several dislocations. These cases had not received a new arthroplasty at the latest follow-up. The last infection occurred after 7 years and was successfully treated with a 2-stage revision with an acceptable clinical outcome. 1 case was surgically treated for heterotopic ossification at 18 months postoperatively. 1 case of dislocation was managed solely by closed reduction and did not experience further complications (Table 5).

**Table 5:** Complications requiring surgical procedures for all 48 cases

Complication	n
Dislocation	2
Infection	
soft tissue debridement	6
implant removed	3
Heterotopic ossification	1

### Implant survival

Revision-free survival of the THA was 89% at 5 years and 74% at 10 years (Figure 3). No revisions were performed later than 7.5 years after the index surgery.

### Mortality

6 cases died within 3 months of the operation, all preoperatively classified as ASA grade 4 by the anesthesiologists. There were no deaths between 3 and 12 months postoperatively, leaving a 1-year mortality rate of 6/48.

## Discussion

We aimed to report on the short- to medium-term outcome of acetabular fractures in 48 cases of osteoporotic bone treated with a combined procedure of ORIF and THA, and attempted to identify factors relevant to patient selection. We found good clinical outcome with a mean HHS of 83 at 1 year after operative in cases with ASA class 1–3 patients but a complication rate of 7/48 and 6 had revision surgery.

Patients included were mainly over 60 years old and had fractures displaying patterns known to have poor results regarding native hip joint survival [4,5,8,17-20]. Most patients (26/48) had anterior column or anterior column posterior hemitransverse fractures, and more than 2 out of 3 had severe joint impactions, previously shown to be more common in fractures in older patients [3,9,21,22].

Our results indicate that most cases with unreconstructable hip joints can be treated with this combined hip procedure with good results. The HHS score in our study was a little lower compared with earlier studies, but these also reported high complication rates of up to 15% [23,24].

Age has been cited as an important risk factor for conversion to THA after ORIF for displaced acetabular fractures [4,7]. A systematic review of the results of operative treatment of acetabular fracture in patients over 55 years reported a 23% conversion rate to THA at a mean 29 months [21]. The results of secondary THA following acetabular fractures are inferior to THA for primary OA [24,25]. Mid-term results have been reported as a HHS of 92 [23]. A recent series of 13 cases reported 100% survival of the THAs at 3 years [26]. The advantages of ORIF and acute THA must be carefully weighed against the risk of severe complications.

An advantage of the combined hip procedure is that it can be applied to all fracture patterns. Other procedures incorporating THA in acute acetabular fracture treatment have been described. In a series of 64 patients treated with acute THA as the sole treatment for their acetabular fracture, over 50% had an HHS of 80 or higher at latest follow-up [11]. A coned hemipelvic acetabular component has been reported with good results in a study of 22 patients, also allowing early weightbearing [27]. Others have also reported good outcome with a variety of adjunct procedures or implants, such as bone impaction grafting, structural bone grafts, reinforcement rings or cages, or augments [13,22,23,26,28]. The large variety in procedures described makes the comparison with a standardized procedure difficult.

The long surgical time in the current study may increase the risk of infection and revision, and may influence the results in these frail patients [29].

We report 89% cumulative survival of the implant at 5 years, comparable to previous reports [13]. 5 of the 6 revisions in our study were performed within 3 months of the index surgery, all due to postoperative infections. Our results clearly indicate that a postoperative infection is catastrophic for these patients; 2 were dead within 3 months of the index operation. In the remaining cases, the implants eventually had to be removed to gain control of the infection. This high risk of early revisions has been previously reported; such as in a series of 22 patients with 5 revisions and in a series of 9 patients with 2 revisions [12,30].

### Strengths and limitations

***Limitations.*** In selecting unreconstructable fractures, the results may not be representative of all geriatric acetabular fractures in a single-center case-series, thus most displaced acetabular fractures in elderly patients should still be treated by conventional ORIF.

***Strengths.*** We used standardization of the surgical procedure, which was performed with standard implants and THA components, applicable to all fracture patterns. As a tertiary referral center, we have treated all available cases in the region consisting of 3.1 million inhabitants.

Based on the findings in the current and previous studies, our institution follows a treatment algorithm for geriatric acetabular fractures (Figure 4).

### Conclusion

Our results indicate that ORIF and acute THA can be performed with good functional results in patients with unreconstructable displaced acetabular fractures, although with a significant risk of infection and revision. Careful patient selection is crucial to reduce the risk of complications and perioperative mortality and thus ASA grade 4 patients should be otherwise treated.

In perspective, acute ORIF and THA is a major surgical procedure with high risk of perioperative complications. Unreconstructable hip joints with comminution and/or joint impaction should be considered for ORIF and acute THA in ASA 1–3 patients. Patients graded ASA 4 are no longer offered this procedure in our department. For such patients, we suggest other treatment options be explored.

## Notes

All authors contibuted in study design and preparation for the study, manuscript writing and manuscript review. RLK: Manuscript preparation, data collection and analysis, statistical analysis. JCJ: Data analysis and statistical analysis.

Handling co-editors: Cecilia Rogmark and Robin Christensen

*Acta* thanks Morten Schultz Larsen and other anonymous reviewers for help with peer review of this manuscript.
